# Representation Matters: Content Analysis of Breastfeeding Images in a Commercial Stock Image Bank

**DOI:** 10.1007/s40615-024-01910-8

**Published:** 2024-01-29

**Authors:** Lauren M. Dinour, Melanie Shefchik

**Affiliations:** 1 Department of Nutrition and Food Studies, Montclair State University, Montclair, NJ USA; 2 Department of Public Health, Montclair State University, Montclair, NJ USA

**Keywords:** Breastfeeding, Content Analysis, Image, Lactation, Skin Color, Representation

## Abstract

Several behavioral change theories posit that normative influences contribute to breastfeeding behaviors and disparities. Given that media has historically presented a narrow view of what is deemed normative in human milk feeding, this study describes who and what is represented in breastfeeding images available in a stock image bank, and whether differences exist based on the breastfeeding parent’s skin color. Using content analysis, the most relevant 2% (*n* = 2284) of breastfeeding and lactation images in Adobe Stock were coded for 60 variables within 12 categories, such as skin color, ability, setting, skin exposure, etc. Descriptive statistics were used to characterize the sample, and the Chi-square test of independence and Mann-Whitney U test were used to compare images of breastfeeding parents with light and non-light skin color. Most images portrayed breastfeeding parents and breastfed children with light colored skin, only one child, an infant-aged child, and no other person. Scant images included accessories considered non-normative. Light skin parents were more frequently depicted with a wedding ring compared to non-light skin parents. Non-light skin parents were more often photographed outdoors compared to light skin parents. Images of light skin parents more frequently showed breast skin, whereas images of non-light skin parents more often showed nipple and/or areola skin. The paucity of diverse people and portrayals of breastfeeding in many ways mirror, and may even perpetuate, societal breastfeeding challenges and inequities. These findings highlight an immediate need for an expanded library of images showcasing a wider variety of breastfeeding experiences.

## Introduction

Human milk is the ideal food for most infants, offering many benefits to infant and parent that alternatives cannot provide [1–3]. For optimal health, the World Health Organization and the American Academy of Pediatrics recommend that infants be exclusively breastfed for the first six months, with continued breastfeeding coupled with complementary foods for at least two years [4, 5]. Yet although most (83.2%) infants born in the United States (US) in 2019 were breastfed at birth, only one-in-four were exclusively breastfed at six months [6]. Additionally, the percent of infants fed any human milk steadily declined with age, from 55.8% at 6 months to 35.9% at 12 months. Even beyond these recommended benchmarks, studies indicate that about 60% of women do not meet personal breastfeeding goals and expectations [7, 8].

In the US, disparities exist in human milk feeding across several socio-demographic characteristics, such as race, maternal education, maternal age, income, and marital status. For example, 74.1% of non-Hispanic Black infants born in 2019 were ever breastfed compared to 90.8% non-Hispanic Asian, 85.3% non-Hispanic white, 83.0% Hispanic, and 82.7% multi-race infants [9]. Similar patterns are seen across breastfeeding duration measures (i.e., 6 months and 12 months) and exclusive breastfeeding time points (i.e., 3 months and 6 months). Breastfeeding rates tend to increase with increasing maternal education levels, age, and income across all outcomes, and infants whose mothers are married have higher rates of ever breastfeeding, exclusive breastfeeding, and breastfeeding at six and 12 months compared to infants of unmarried mothers [9].

Several behavioral change theories and models, such as the Theory of Planned Behavior [10], Integrated Behavioral Model [11], Social Cognitive Theory [12], and Social Norms Approach [13] posit that health behaviors—like human milk feeding—are driven in part by normative influences. Media contributes to these norms by reaching wide audiences with the potential to influence viewer/consumer beliefs regarding the acceptability of depictions. Magazines, articles, television, and campaigns can shape attitudes towards breastfeeding depending on the content’s nature, and even hinder breastfeeding efforts by eliciting feelings of discomfort, embarrassment, or disapproval [14–17]. For instance, research on the frequency of advertisements depicting alternatives to human milk in *Parents’ Magazine* found that, as these increased, breastfeeding rates in US women declined the following year [18]. Such results suggest that increased promotion of human milk alternatives in a popular magazine is associated with national changes in breastfeeding decisions.

Breastfeeding representation in media presents a particularly narrow story for what is deemed normative. For example, analysis of fictional television indicates the dominance of breastfeeding characters who fit into the narrative of “professional, affluent, well educated, and usually Caucasian” women [19]. In the few examples that deviate from this norm, positive experiences and visual portrayals of breastfeeding are absent [19]. Even in educational contexts on social media, videos predominantly appeal to white populations with the highest rates of breastfeeding, while missing the opportunity to represent and target populations who may benefit most, especially women of color and indigenous women [20]. This may contribute to in-group bias and injunctive norms within minority communities, especially without infinite examples of role models [21, 22].

What is unclear is whether media outlets intentionally narrow breastfeeding representation, or whether diverse images are simply unavailable. The purpose of this study is to examine who and what is represented in breastfeeding images available from a stock image bank, often a source of images used in print and digital media. Given that prior media-based research has focused on race, ethnicity, and class depictions of breastfeeding, we sought to analyze additional forms of representation, such as child’s age, presence of tattoos and piercings, ability, etc. We present results of our content analysis of a sample of images from Adobe Stock, a popular international stock image bank offering over 200 million photos [23]. Additionally, we explore whether presence of these additional forms of representation differs by the breastfeeding parent’s skin color.

## Methods

### Sample

On June 3, 2022, we searched Adobe Stock [24] for “breastfeeding” and “lactation,” resulting in 331,090 hits. Filtering options were set to select the asset type (i.e., images only, no videos or templates), subcategory (i.e., photos only, no illustrations or vectors), price (i.e., standard content only, no premium content), and people (i.e., include people in the image). After applying filters, 114,203 images remained. We used the sorting feature to order images by relevance and saved the first 2% (*n* = 2284) to an Adobe Stock library.

Image metadata (i.e., identification number, title, category, uploader, and keywords) were electronically copied to a spreadsheet. Image identification numbers were randomized and split into three Google Sheets [25], each containing two-thirds (*n* = 1523) of the sample so that every image could be independently coded by two coders. During the coding process, 26 images were deemed not appropriate for analysis because they portrayed elderly adults with or without assistance from a healthcare professional (*n* = 19), healthcare professionals alone (*n* = 3), school-aged children engaged in unrelated activities (*n* = 3), or a knitted doll (*n* = 1).

**Fig. 1:**
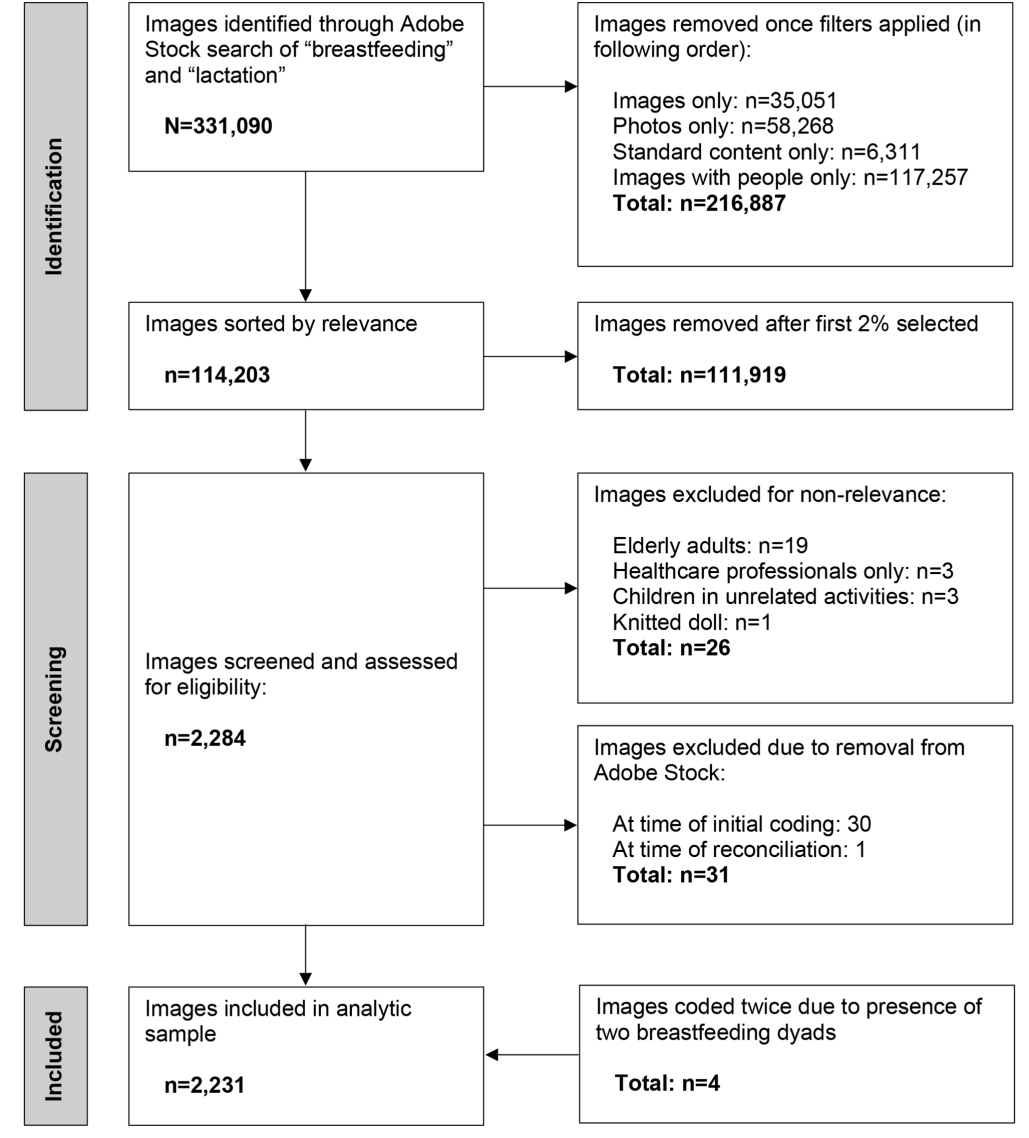
Flow chart of image sample selection

Additionally, 30 images were no longer available at the time of initial coding, and one was no longer available during the coding reconciliation period. According to Adobe Stock, images may be deleted by the uploader [26] or removed by Adobe for any reason [27]. Although we were unable to view the withdrawn images to consider reasons for removal, review of image keywords did not reveal any patterns. These images were subsequently removed from the sample. Finally, four images each depicted two breastfeeding dyads. Given our goal of understanding breastfeeding representation, we treated each image as if it was two separate images (i.e., one set of codes for each breastfeeding dyad)—for a final analytic sample of 2231 images (Fig. 1).

### Measures

Given that breastfeeding disparities exist by race/ethnicity, we were interested in identifying whether similar patterns were reflected in the image sample. However, presuming race/ethnicity from an image is problematic as it assumes that individuals present similarly. Yet race is increasingly recognized as a social rather than a biological construct [28, 29]. To address this, we measured skin color using the Project on Ethnicity and Race in Latin American (PERLA) color palette [30]. The palette consists of 11 skin tones, with 1 being the lightest and 11 the darkest. For the current study, skin colors of both the breastfeeding parent and breastfed child(ren) were assessed with a printed and laminated copy of the PERLA color palette.

To determine other forms of representation, a coding schema was created for additional categories reflecting who and what is portrayed in each image. Specific variables and codes within these categories are outlined in Table 1.

**Table 1:** Breastfeeding image coding schema with categories, variables, and codes

Category	Variable	Codes
Image ID #	Image ID #	Pre-populated
Skin Color	Skin color # of BF parent	Insert 1-11 96 = Image is in B&W 97 = No skin showing 98 = No BF parent in image
	Skin color # of BF child(ren)	Insert 1-11 96 = Image is in B&W 97 = No skin showing 98 = No BF child in image
Number of Children Being Breastfed	Number of children being breastfed	Insert #
	If > 1, do the BF children appear to be twins/multiples?	0 = No 1 = Yes 98 = N/A
Life Stage of Child(ren) Being Breastfed	Infant (< 12 months)	0 = No 1 = Yes 97 = Child not visible 98 = No child present
	Toddler (1–3 years)	0 = No 1 = Yes 97 = Child not visible 98 = No child present
	School-aged child (4 + years)	0 = No 1 = Yes 97 = Child not visible 98 = No child present
People in the Image	Is BF parent looking at the BF child?	0 = No 1 = Yes 97 = BF parent’s head not visible 98 = No BF child and/or BF parent
	How many other people are in the image besides BF parent and BF child?	Insert #
	Who else is in the image?	0 = No one else 1 = Partner of different sex 2 = Partner of same sex 3 = Another child 4 = Grandparent 5 = Friend/other relative 6 = Health professional 7 = Can’t tell, full body not visible
	Is the BF parent and/or partner wearing a wedding ring?	0 = No 1 = Yes 2 = Hands not visible
	Does the BF parent have any visible piercings beyond the ear lobe?	0 = No 1 = Yes
	Does the BF parent have one or more visible tattoos?	0 = No 1 = Yes
	Is anyone in the image wearing a face mask?	0 = No 1 = Yes, the BF parent is 2 = Yes, someone else is
Ability	Are there any assistive equipment present (e.g., walker, cane, wheelchair, hearing aid, glasses, etc.)?	0 = No 1 = Yes
	What assistive equipment are present, and who is using the equipment?	Specify
Facial Expression of BF Parent	Face visible?	0 = No 1 = Yes 98 = No BF parent
	Smile	0 = No 1 = Yes 97 = Face not visible 98 = No BF parent
	Grimace	0 = No 1 = Yes 97 = Face not visible 98 = No BF parent
	Frown	0 = No 1 = Yes 97 = Face not visible 98 = No BF parent
	Yawn/tired	0 = No 1 = Yes 97 = Face not visible 98 = No BF parent
	Flat	0 = No 1 = Yes 97 = Face not visible 98 = No BF parent
	Crying	0 = No 1 = Yes 97 = Face not visible 98 = No BF parent
Facial Expression of BF Child	Face visible?	0 = No 1 = Yes 98 = No BF child
	Crying/distress	0 = No 1 = Yes 97 = Face not visible 98 = No BF child
	Smile/happy	0 = No 1 = Yes 97 = Face not visible 98 = No BF child
	Sleepy/sleeping	0 = No 1 = Yes 97 = Face not visible 98 = No BF child
	Alert w/o expression	0 = No 1 = Yes 97 = Face not visible 98 = No BF child
	Is BF child currently being fed at the breast?	0 = No 1 = Yes 98 = No BF child and/or BF parent
Setting	Home	0 = No 1 = Yes
	Hospital or clinical setting	0 = No 1 = Yes
	Vehicle	0 = No 1 = Yes
	Restaurant	0 = No 1 = Yes
	Office/work	0 = No 1 = Yes
	Photo studio or nondescript background	0 = No 1 = Yes
	Outdoors	0 = No 1 = Yes
	If outdoors, describe setting	Specify
	Other	Specify
Skin Exposure of BF Parent	Neck and/or chest (above breasts) skin showing	0 = No 1 = Yes 98 = No BF parent
	Stomach skin showing	0 = No 1 = Yes 98 = No BF parent
	Breast skin showing (not nipple or areola)	0 = No 1 = Yes 98 = No BF parent
	Nipple and/or areola showing	0 = No 1 = Yes 98 = No BF parent
Other Activities of BF Parent Occurring Simultaneously with BF	Eating	0 = No 1 = Yes
	Drinking	0 = No 1 = Yes
	Working	0 = No 1 = Yes
	Watching TV	0 = No 1 = Yes
	Looking at phone	0 = No 1 = Yes
	Tending to another child	0 = No 1 = Yes
	Any other activities?	0 = No 1 = Yes
	Other	Specify
Presence of BF/Feeding Equipment	Bottles	0 = No 1 = Yes
	Breast pump	0 = No 1 = Yes
	Nipple shield	0 = No 1 = Yes
	Supplemental nursing system	0 = No 1 = Yes
	BF pillows	0 = No 1 = Yes
	Infant formula	0 = No 1 = Yes
	Educational model (e.g., baby doll, breast)	0 = No 1 = Yes
	Nursing cover	0 = No 1 = Yes
	Other	Specify
Additional Notes	Specify any outliers (e.g., age of BF parent < 20 or > 50; gender of BF parent not woman; religious or ethnic clothing; amputations/missing limbs; etc.)	Specify

*ID* identification, *BF* breastfeeding, *B&W* black and white

### Data Collection

We trained three undergraduate students to code images using the coding schema. Attempts were made at recruiting a diverse set of coders to reduce bias in coding. However, all three coders identified as undergraduate students, women, and white. One coder identified as Hispanic and Spanish-speaking, while the other two identified as non-Hispanic, English-only speakers. Coders attended two, two-hour trainings and practiced coding images between sessions. Instructions given for coding skin color included viewing all images on a computer in a well-lit indoor space with the screen set to full brightness. Coding occurred between September and December 2022.

Coders were asked to look only at the image and not at any other data, such as the title or keywords. Once all initial coding was complete, spreadsheets were combined, and codes were compared for each image. Percent agreement was calculated for each variable, with 51 of 60 variables achieving > 90% agreement and an additional seven variables ≥ 83%. Agreement was lowest for skin color of breastfeeding parent (60%) and child (59%). Although low, studies of similar skin color palettes have observed percent agreement of only 25–33% [31]. As well, most (87%) discrepancies were within one point, and only 2% were within three or four points. A plurality (93%) of discrepancies occurred within the lightest three skin tones. When a discrepancy was noted between coders, the third coder was asked to code the discrepant variable(s). At this stage, at least two coders agreed on the code at least 98% of the time (variables ranged from 98 to 100% agreement).

To create the final dataset, any variable where the original two coders agreed was assigned that code. For variables where there were disagreements, the third coder’s assessment determined the final code only if there was agreement with one of the original coders. Following the third coder’s assessment, 388 instances (out of 132,422 ratings, or 0.3%) remained where we did not have at least two coders agree, and 59% of these (*n* = 227) were due to only one of the three coders entering a code (either because of an unintentional skipping of a cell or a lack of qualitative specification). Forty-one (11%) of the discrepant cases occurred in a write-in variable (e.g., Outdoor-specify or Other-specify) and were reconciled by one of the original coders based on similarity in meaning. Additionally, 16% (*n* = 62) of the discrepant cases were found in the skin color variables. Due to the subjective nature of the skin color assessment, for the two skin color variables we used the average of the three codes in the final dataset rather than solicit a fourth coder. For all other variables with less than two coders in agreement (*n* = 285), the two lead researchers agreed upon the final code.

### Data Analysis

The final dataset was uploaded to SPSS Version 27 [32] for quantitative analysis. Two continuous variables were created: (1) *breastfeeding dyad skin color difference* was calculated by subtracting the skin color score of the breastfed child from the breastfeeding parent (possible range of -10 to + 10), and (2) *skin exposure scale* was calculated by summing the areas of the breastfeeding parent’s skin exposure (possible range of 0 to 4).

Descriptive statistics were used to characterize the sample and are reported as frequencies and percentages. The two continuous skin color variables (potential range: 1–11) had a skewed distribution, such that only 14% of breastfeeding parents and breastfed children were coded above 3. To make comparisons, each variable was categorized using the delineations specified by Telles et al. [33] and modified to account for the averages of discrepant codes: light skin (1-3.4), medium skin (3.5–5.4), and dark skin (5.5–11). However, this categorization did not yield adequate numbers in the medium and dark skin groups for statistical analysis. Therefore, all comparisons were made between light skin (1-3.4) and non-light skin (3.5–11) groups.

The Chi-square test of independence was used to compare the categorical characteristics between images of breastfeeding parents with light and non-light skin. Cramer’s V was calculated to determine the strength of the association between categorical variables. For continuous variables, the Shapiro-Wilk test was used to test for normality. Because the data were not normally distributed, the Mann-Whitney U test was used to compare the difference between images of breastfeeding parents with light and non-light skin. Median and interquartile range values are reported. All tests were two-tailed, and significance was defined at *P* < 0.05.

## Results

### Aim 1: Description of Who and What is Represented in Breastfeeding Images

The sample was composed of 2231 images, of which 97% (*n* = 2167) included a breastfeeding parent and 95% (*n* = 2127) included a breastfed child who could be coded for skin color. The average skin color score was 2.0 +/- 0.91 for breastfeeding parents and 1.7 +/- 1.00 for breastfed children. In both cases, 96% of the sample that could be coded for skin color was coded in the light skin category (1-3.4), 2–3% in the medium skin category (3.5–5.4), and 1% in the dark skin category (5.5–11). Although the skin color scale ranged from 1 to 11, no breastfeeding parent was coded above 9, and only one breastfed child was coded at 10 with none coded 11.

About half of the images (53%, *n* = 1114) that could be coded for skin color included a breastfeeding parent and breastfed child with the same skin color score. Another 45% (*n* = 938) of images showed a breastfeeding parent and breastfed child with skin colors differing by no more than one point. The average difference in skin color between parent and child was 0.3 +/- 0.69 (range − 3.0 to 3.0), meaning that breastfeeding parents had slightly darker skin then their breastfed children, on average. When looking at the absolute difference, breastfeeding parents and children had skin colors that were an average of 0.5 +/- 0.56 points apart (range 0 to 3.0).

A plurality of images included only one breastfed child (97%), an infant-aged child being breastfed (87%), and no other person in the photo (95%). Only 14 images (0.6%) depicted tandem breastfeeding, and only six images (0.3%) showed a school-aged child breastfeeding. Of the 103 images that included someone else in the photo, 41 (40%) depicted an adult who appeared to be a partner, only one of which was of the same sex. Although marital status cannot be confirmed, among the 619 images where hands were visible, 62% (*n* = 381) showed the breastfeeding parent and/or perceived partner wearing a wedding ring.

Scant images included accessories that could be considered non-normative. For example, only two (0.1%) showed a breastfeeding parent with piercings in a location other than the earlobe, while four (0.2%) included a breastfeeding parent with tattoos. Fifteen images (0.7%) included someone wearing a face mask, half of whom (*n* = 7) were the breastfeeding parent. Regarding ability, 16 images (0.7%) depicted assistive equipment, though only eyeglasses (*n* = 13) and a wrist brace (*n* = 3) were shown.

Facial expressions of breastfeeding parents and children varied, though most images included positive or neutral expressions. For example, of the 1361 images where the breastfeeding parent’s face was visible, 60% (*n* = 813) were smiling and 37% (*n* = 509) had a flat expression. The remaining 3% of images depicted expressions of tiredness (*n* = 22), grimacing (*n* = 9), or frowning (*n* = 9). Among the 1547 images with a visible breastfed child’s face, the breastfed child(ren) was alert without any expression in 59% (*n* = 918), sleepy or sleeping in 37% (*n* = 577), and smiling in 3% (*n* = 46). Ten images (0.6%) show a breastfed child crying or in distress.

Images were taken in several settings, most frequently in a photo studio or non-descript space (58%) or at home (30%). Fewer images were taken in public settings like outdoor locations (8%), restaurants (0.5%), or shopping malls (0.4%). Additionally, in nearly all images, the breastfeeding parent was not engaged in any other activity besides breastfeeding. Only 4% of images depicted a breastfeeding parent multi-tasking in some way, such as using their phone, working, drinking, eating, or tending to another child.

Regarding skin exposure, of the 2212 images that included a breastfeeding parent, 86% (*n* = 1903) showed skin in the neck and/or chest (above breasts) area, while 82% (*n* = 1812) showed breast skin. Nipples and/or areolas were viewable in 40% (*n* = 877) of images, with only 13% (*n* = 284) revealing stomach skin. The number of areas of skin exposure was summed (range: 0–4), with a plurality of images revealing two (45%) or three (32%) areas of skin. Only 5% (*n* = 111) of images included all four areas of skin exposure, slightly higher than the 3% (*n* = 75) of images with no skin exposure. Among the 2183 images that included at least one breastfed child, 88% (*n* = 1925) showed the child being fed at the breast. Additionally, 6% (*n* = 145) of images included at least one breastfeeding equipment item, such as a breast pump, bottle, breastfeeding pillow, etc. Additional image characteristics are detailed in Table 2.

**Table 2:** Descriptive characteristics of breastfeeding images (*N* = 2231)

Characteristic	Mean	SD
**Skin color of BF parent** (*n* = 2167)^a^	2.0	0.91
**Skin color of BF child(ren)** (*n* = 2127)^a^	1.7	1.00
**BF dyad skin color difference** (*n* = 2109)^b^	0.29	0.69
**BF dyad skin color difference (absolute)** (*n* = 2109)^b^	0.50	0.56
	**Frequency**	**Percent** ^**c**^
**BF parent skin color category**		
Light (1-3.4)	2072	92.9%
Medium (3.5–5.4)	64	2.9%
Dark (5.5–11)	31	1.4%
Image not in color, no skin showing, or no BF parent	64	2.9%
**BF child skin color category**		
Light (1-3.4)	2050	91.9%
Medium (3.5–5.4)	48	2.2%
Dark (5.5–11)	29	1.3%
Image not in color, no skin showing, or no BF child	104	4.7%
**BF dyad skin color difference (absolute)**		
0	1114	49.9%
> 0 to 1	938	42.0%
> 1 to 2	51	2.3%
> 2 to 3	6	0.27%
Image could not be coded for skin color	122	5.5%
**Number of BF children**		
0	38	1.7%
1	2173	97.4%
2	20	0.90%
**If > 1 BF child, do children appear as multiples?**		
Yes	15	0.63%
No	5	0.22%
N/A	2211	99.1%
**BF child(ren) lifestage** ^**d**^		
Infant (< 12 months)	1938	86.9%
Toddler (1–3 years)	249	11.2%
School aged (4 + years) BF child not visible or present	6 43	0.27% 1.9%
**Is BF parent looking at the BF child?**		
Yes	1274	57.1%
No	300	13.4%
BF parent’s head not visible or no BF child or parent	657	29.4%
**Number of other people in image**		
0	2128	95.4%
1	88	3.9%
2	15	0.67%
**Who else is in the image?**		
Partner of different sex	37	1.7%
Partner of same sex	1	0.04%
Another child(ren)	25	1.1%
Grandparent	1	0.04%
Friend/other relative	5	0.22%
Health professional	18	0.81%
Partner of different sex and another child	3	0.13%
Another BF dyad	8	0.36%
Can’t tell, full body not visible	5	0.22%
No one else	2128	95.4%
**Is BF parent and/or partner wearing wedding ring?**		
Yes	381	17.1%
No	238	10.7%
Hands not visible	1612	72.3%
**Does BF parent have piercings beyond ear lobe?**		
Yes No	2 2229	0.09% 99.9%
**Does BF parent have** $\ge$ **1 visible tattoos?**		
Yes	4	0.18%
No	2227	99.8%
**Is anyone in the image wearing a face mask?**		
Yes, the BF parent is	7	0.31%
Yes, someone else in the image is	8	0.36%
No	2216	99.3%
**Assistive equipment present in image**		
Glasses on BF parent	7	0.31%
Glasses on someone other than BF parent Wrist brace on BF parent	4 3	0.18% 0.13%
Glasses on the table	1	0.04%
Glasses on BF parent and someone else	1	0.04%
None	2215	99.3%
**BF parent facial expression**		
Smile	813	36.4%
Grimace	9	0.40%
Frown	9	0.40%
Yawn/tired	22	0.99%
Flat	508	22.8%
Crying	0	0.0%
Face not visible	870	39.0%
**BF child facial expression** ^**e**^		
Crying/distress	10	0.45%
Smile/happy	46	2.1%
Sleepy/sleeping	577	25.9%
Alert w/o expression	918	41.1%
Face not visible	684	30.7%
**Is BF child currently being fed at the breast?**		
Yes	1925	86.3%
No	258	11.6%
No BF child and/or BF parent in image	48	2.2%
**Setting**		
Home	667	29.9%
Hospital or clinical setting	65	2.9%
Vehicle	6	0.3%
Restaurant	11	0.49%
Office/work	4	0.18%
Photo studio or nondescript background	1300	58.3%
Mall	8	0.36%
Indoor pool	1	0.04%
Outdoors	169	7.6%
**Outdoor settings specified**		
Park	68	3.0%
Field Yard or outside home	44 33	2.0% 1.5%
Beach	14	0.63%
Public street/plaza	10	0.45%
N/A	2062	92.4%
**Areas of BF parent skin exposure** ^**d**^		
Neck and/or chest (above breasts)	1903	85.3%
Stomach	284	12.7%
Breast (not nipple or areola)	1812	81.2%
Nipple and/or areola	877	39.3%
No BF parent in image	19	0.85%
**Number of areas of BF parent skin exposure**		
None of the areas of skin showing	75	3.4%
1 area of skin showing	327	14.7%
2 areas of skin showing	992	44.5%
3 areas of skin showing	707	31.7%
4 areas of skin showing	111	5.0%
No BF parent in image	19	0.85%
**BF parent doing other activities while BF** ^**f**^		
Eating	5	0.22%
Drinking	12	0.54%
Working	23	1.0%
Watching TV	0	0.0%
Looking at phone	24	1.1%
Tending to another child	6	0.27%
Talking to someone (in-person, voice call, video call)	13	0.58%
Using or cleaning breast pump	9	0.40%
Taking photo or video	4	0.18%
Looking at clothing/object	3	0.13%
Reading	2	0.09%
Sleeping/napping	1	0.04%
Meditating	1	0.04%
No other activities	2132	95.6%
**BF or feeding equipment in image**		
Bottles	44	2.0%
Breast pump	52	2.3%
Nipple shield Supplemental nursing system	4 1	0.18% 0.04%
BF pillows	32	1.4%
Infant formula	0	0.0%
Educational model	6	0.27%
Nursing cover	8	0.36%
Nursing bra/shirt	12	0.54%
Baby carrier	3	0.13%
Breast milk storage bags	2	0.09%
Milk saver collector	1	0.04%
None	2086	93.5%
**Outliers**		
BF parent wearing ethnic head covering	8	0.36%
BF parent wearing hijab	6	0.27%
BF parent’s hair (short, dread locs, box braids)	3	0.13%
BF child has bald spots	3	0.13%
BF parent wearing other religious or ethnic items	2	0.09%
BF child has pierced ears	1	0.04%
BF parent holding beer	1	0.04%
None	2207	98.9%

*SD* standard deviation, *BF* breastfeeding, *N/A*, not applicable

^a^Not all images in the sample included a breastfeeding parent and/or a breastfeeding child

^b^The breastfeeding dyad skin color difference was calculated by subtracting the skin color score of the breastfed child from the breastfeeding parent (possible range of -10 to + 10) and is based on images where both a breastfeeding parent and a breastfeeding child are present/visible (excluding black and white images)

^c^Denominator is total images in sample (*N* = 2231)

^d^Categories are not mutually exclusive

^e^Numbers exceed sample size because four images included two breastfeeding children who showed different facial expressions

^f^The following breastfeeding parent activities are not mutually exclusive: looking at phone and using or cleaning breast pump; working/on laptop and talking to someone else

### Aim 2: Comparison of Image Characteristics by Skin Color Score of Breastfeeding Parents

Several significant differences were noted between images of breastfeeding parents with light skin (skin color score of 1-3.4) and non-light skin (skin color score of 3.5–11) (Table 3). For example, a larger percentage of non-light skin parents had skin colors that differed from their breastfed child by more than one point compared to light skin parents (17% vs. 2%, *p* < 0.001). However, no significant difference was noted for breastfeeding dyad skin color difference when treated as a continuous variable (Table 4). Light skin parents more frequently wore a wedding ring compared to non-light skin parents (64% vs. 23%, *p* < 0.001, Table 3). In terms of image setting, non-light skin parents were more often photographed outdoors compared to light skin parents (14.7% vs. 7.5%, *p* = 0.01), though no significant differences were found in any other setting type.

**Table 3:** Comparison of image characteristics between images with light skin and non-light skin breastfeeding parents (*n* = 2167)

Characteristic	Light Skin Parent^a^, n (%)	Non-Light Skin Parent^b^, n (%)	*P*
**BF dyad skin color difference > 1 point** ^**c**^	41 (2.0)	16 (17.0)	< 0.001***
**BF child(ren) lifestage**			
Infant (< 12 months)	1798 (88.6)	81 (86.2)	0.48
Toddler (1–3 years)	231 (11.4)	13 (13.8)	0.47
School aged (4 + years)	6 (0.3)	0 (0.0)	0.60
**BF parent is looking at the BF child**	1198 (95.5)	281 (94.9)	0.70
**Wedding ring on BF parent and/or partner**	373 (63.5)	5 (22.7)	< 0.001***
**Assistive equipment present in image**	16 (0.8)	0 (0.0)	0.39
**BF parent facial expression**			
Smile	762 (59.8)	41 (61.2)	0.82
Grimace	9 (0.7)	0 (0.0)	0.49
Frown	9 (0.7)	0 (0.0)	0.49
Yawn/Tired	20 (1.6)	1 (1.5)	0.96
Flat	475 (37.3)	25 (37.3)	0.99
**Setting**			
Home	633 (30.6)	26 (27.4)	0.51
Hospital or clinical setting	62 (3.0)	3 (3.2)	0.93
Vehicle	5 (0.2)	1 (1.1)	0.14
Restaurant	11 (0.5)	0 (0.0)	0.48
Office/work	4 (0.2)	0 (0.0)	0.67
Photo studio or nondescript	1193 (57.6)	51 (53.7)	0.45
Outdoors	155 (7.5)	14 (14.7)	0.01*
**Areas of BF parent skin exposure**			
Neck and/or chest (above breasts)	1788 (86.3)	81 (85.3)	0.78
Stomach	266 (12.8)	12 (12.6)	0.95
Breast (not nipple or areola)	1702 (82.1)	66 (69.5)	0.002**
Nipple and/or areola	803 (38.8)	48 (50.5)	0.022*
3 or 4 areas of skin exposure^d^	748 (36.1)	48 (50.5)	0.004**
**BF parent doing other activities while BF**			
Any other activities	96 (4.6)	2 (2.1)	0.25
Working or on laptop	23 (1.1)	1 (1.1)	0.96
Looking at phone	26 (1.3)	0 (0.0)	0.27
**Any BF or feeding equipment in image**	129 (6.2)	8 (8.4)	0.39

*BF* breastfeeding

^a^Light skin is defined as a skin color score of 1-3.4 (*n* = 2072)

^b^Non-light skin is defined as a skin color score of 3.5–11 (*n* = 95)

^c^The breastfeeding dyad skin color difference was calculated by subtracting the skin color score of the breastfed child from the breastfeeding parent (possible range of -10 to + 10)

^d^Based on skin exposure scale, calculated by summing the number of areas of the breastfeeding parent’s skin exposure (possible range of 0 to 4)

*P < 0.05; **P < 0.01; ***P < 0.001

**Table 4:** Comparison of continuous variables between images with light skin and non-light skin breastfeeding parents (*n* = 2167)

Characteristics	Light Skin Parent^a^, Mean (IQR)	Non-Light Skin Parent^b^, Mean (IQR)	U	z	*P*
BF dyad skin color difference^c^	0.0 (0.0–1.0)	0.3 (0.0–1.0)	87216.0	-1.445	0.15
Number of BF children	1.0 (1.0–1.0)	1.0 (1.0–1.0)	96462.5	-1.184	0.24
Skin exposure scale^d^	2.0 (2.0–3.0)	3.0 (1.0–3.0)	95684.0	-0.491	0.62

*IQR* interquartile range, *U* Mann-Whitney test, *z* Mann-Whitney score, *BF* breastfeeding

^a^Light skin is defined as a skin color score of 1-3.4 (*n* = 2072)

^b^Non-light skin is defined as a skin color score of 3.5–11 (*n* = 95)

^c^The breastfeeding dyad skin color difference was calculated by subtracting the skin color score of the breastfed child from the breastfeeding parent (possible range of -10 to + 10)

^d^The skin exposure scale was calculated by summing the number of areas of the breastfeeding parent’s skin exposure (possible range of 0 to 4)

*P < 0.05; **P < 0.01; ***P < 0.001

Differences were also noted regarding skin exposure (Table 3). Compared to images of non-light skin breastfeeding parents, images of light skin parents more frequently showed breast skin (70% vs. 82%, *p* = 0.002). Conversely, images of non-light skin breastfeeding parents more often showed nipple and/or areola skin compared to images of light skin parents (51% vs. 39%, *p* = 0.02). Additionally, images of non-light skin breastfeeding parents more frequently included three or all four areas of skin exposure compared to light skin parents (51% vs. 36%, *p* = 0.004). No significant differences between groups were noted for the continuous skin exposure scale (Table 4).

Groups did not differ in terms of the breastfeeding child’s life stage, breastfeeding parent looking at the breastfed child, parent’s facial expression, other activities occurring in the image, presence of breastfeeding equipment or assistive devices (Table 3), or number of breastfed children (Table 4).

## Discussion

Images are powerful tools that can support information dissemination, reinforce messages, evoke emotions, and influence behaviors [34, 35]. Images shape sociocultural norms and contribute to media representations of breastfeeding, both of which influence infant feeding decisions [36]. Our goal was to explore the availability of images that could be used for commercial, media, educational, or health promotion purposes. Results from this content analysis indicate homogeneity among breastfeeding-related images found in a large commercial image bank. Images overwhelmingly illustrated lighter-skinned, able-bodied, married people breastfeeding infant-aged children in private spaces. This paucity of diverse people and portrayals of breastfeeding in many ways mirror societal breastfeeding challenges and inequities.

Our results are consistent with that of Foss [19], who examined television depictions of breastfeeding and found that the breastfeeding woman is represented as professional, affluent, well-educated, and usually Caucasian. A decade has passed since this publication, yet our results reveal a lack of progress on illustrating diverse breastfeeding experiences. Like Foss, we found breastfeeding images to predominantly feature able-bodied and heterosexual people with lighter skin color. Other recent studies that have explored gender, sexual orientation, ableness, and racial diversity among images used in midwifery and human sexuality textbooks and outdoor magazines show similar findings [37–39]. This lack of diversity reinforces “typical norms” and harmful societal narratives.

Images that did depict medium and dark skin parents were more likely to include nipple and areola exposure and have more skin areas exposed than light skin parents. Notably, Villalobos, et al. [40] described perceptions of stigma, fear, and shame for nursing in public, with concerns related to modesty amongst African American mothers. Thus, findings from our study may conflict with the community’s injunctive norms. While we support the normalization of skin exposure for breastfeeding, if breastfeeding disparities are to be addressed, then images must be relevant and culturally acceptable. Our study did not analyze the photographers to determine if they reflect the communities they photograph, which may exacerbate this misalignment.

Our findings illustrate a lack of images of individuals breastfeeding in the presence of other people and in a variety of social circumstances. Despite societal efforts to normalize breastfeeding, only 12% of images in our sample were in public settings (restaurants, offices, malls, pools) and only 5% of images included another person in the photo. The most common individuals illustrated in the photo, besides the breastfeeding person or child, were perceived partners, another child, or a health professional. In a commentary exploring breastfeeding in recent photography, Giles [41] notes a reluctance to shift from understanding breastfeeding as a solitary activity to a companionable behavior embedded in the social landscape. A wider variety of images might encourage individuals to breastfeed openly in many societal settings, supporting enhanced breastfeeding duration and exclusivity.

Furthermore, images may not reflect the current realities and variations in breastfeeding experiences. Very few images showed expressions of tiredness, grimaces, or frowning on the breastfeeding parent, multi-tasking of activities, or breastfeeding supplies and equipment. When using images for information dissemination and health promotion, it is important to select realistic and relatable portrayals, showcasing variety in experiences and the positive, negative, and neutral aspects of the behavior [34]. Meeting this recommendation may be challenged by current availability of images.

### Strengths

This study had several strengths. First, we analyzed images available in one of the largest international image banks with more than 200 million images [23]. Another strength is the analysis of a large sample of images sorted by relevance, which is consistent with what the user would find when searching for breastfeeding or lactation images on this platform. In this study, we coded for identifiers and characteristics not included in previous studies. This study also utilized a novel approach to coding images using the PERLA color palette [30], which allowed for objectivity and a wider range of skin color representations to be analyzed. Finally, the high levels of inter-rater reliability achieved across all variables instills confidence in study findings.

### Limitations

A limitation of this study is that only one image bank was searched. Despite analyzing a commonly-used, large image bank [23], the findings may not be generalizable to other commercial stock photography venues or represent the full scope of available images. Additionally, we used a cross-sectional design where images were searched at a single time point. Thus, the availability of images and their order by relevance may change over time. Despite our attempts to recruit a diverse research team, none of our coders identified as male, African American or Black, American Indian or Alaskan Native, Asian, Native Hawaiian or Pacific Islander, or other. While this could have influenced the analysis, we trained all coders to use the PERLA color palette [30] rather than make subjective assumptions regarding skin color, race, or ethnicity in order to reduce bias. Finally, given that a small proportion of images illustrated a breastfeeding parent with non-light skin, statistical comparisons could not be made for many variables due to lack of adequate variation.

### Implications for Practice

Thoughtful image use can challenge assumptions and change harmful narratives that perpetuate breastfeeding inequities. While it is important to encourage use of images that are authentic, accurate, and respectful, these intentions are limited by what is available. These findings highlight a need for an expanded library of diverse breastfeeding images. Recently, non-profits have attempted to fill these gaps. For example, the U.S. Breastfeeding Committee established the “Landscape of Breastfeeding Support” gallery, which contains more than 10,000 high quality images illustrating how communities can support breastfeeding [42]. Aiming to undo implicit bias in medical images and normalize how breast conditions manifest in patients of color, the Melanated Mammary Atlas is a searchable collection of images illustrating various breast-related conditions on brown skin [43]. This directory is accessible to verified health professionals and students. However, a need persists within commercial image banks as these are commonly used in education and mass media.

Organizing professional, high-quality photo shoots to capture breastfeeding with diverse people, places, and experiences is a necessary next step towards improvement. Commercial image banks, including Adobe Stock, are user-submitted repositories and thus opportunities exist to enhance their offerings. Similarly, breastfeeding images are lacking in non-commercial sources. For instance, the Centers for Disease Control and Prevention’s public health image library contains only two breastfeeding images as of this writing [44]. Efforts can therefore be made by governmental image banks to expand their selection of images, as these may also be common venues for public health agencies seeking copyright-free images.

## Conclusion

Lack of diversity in images can reinforce assumptions about who typically breastfeeds and may perpetuate existing disparities. Richer, diverse, and more holistic representations of breastfeeding are needed in commercial stock photography.

## Data Availability

Not applicable.

## References

[1] Binns C, Lee M, Low WY. The long-term public health benefits of breastfeeding. Asia Pac J Public Health. 2016;28:7–14. 10.1177/1010539515624964. 26792873 10.1177/1010539515624964

[2] Dieterich CM, Felice JP, O’ Sullivan E, Rasmussen KM. Breastfeeding and health outcomes for the mother-infant dyad. Pediatr Clin North Am. 2013;60:31–48. 10.1016/j.pcl.2012.09.010. 23178059 10.1016/j.pcl.2012.09.010 PMC3508512

[3] Ip S, Chung M, Raman G, Trikalinos TA, Lau J. A summary of the Agency for Healthcare Research and Quality’s evidence report on breastfeeding in developed countries. Breastfeed Med. 2009;4(Suppl 1):17–30. 10.1089/bfm.2009.0050. 19827919 10.1089/bfm.2009.0050

[4] Meek JY, Noble L, Section on Breastfeeding. Policy statement: breastfeeding and the use of human milk. Pediatrics. 2022;150:e2022057988. 10.1542/peds.2022-057988. 35921640 10.1542/peds.2022-057988

[5] World Health Organization/United Nations Children’s Fund. Global Strategy for Infant and young child feeding. Geneva: World Health Organization; 2003.

[6] Centers for Disease Control and Prevention. Breastfeeding Report Card United States, 2022 [Internet]. 2022. Available from: https://www.cdc.gov/breastfeeding/data/reportcard.htm.

[7] Gregory EF, Butz AM, Ghazarian SR, Gross SM, Johnson SB. Are unmet breastfeeding expectations associated with maternal depressive symptoms? Acad Pediatr. 2015;15:319–25. 10.1016/j.acap.2014.12.003. 25906701 10.1016/j.acap.2014.12.003

[8] Odom EC, Li R, Scanlon KS, Perrine CG, Grummer-Strawn L. Reasons for earlier than desired cessation of breastfeeding. Pediatrics. 2013;131:e726–732. 10.1542/peds.2012-1295. 23420922 10.1542/peds.2012-1295 PMC4861949

[9] Centers for Disease Control and Prevention. National Immunization Survey (NIS) Results: Breastfeeding Rates [Internet]. 2022 [cited 2023 Feb 8]. Available from: https://www.cdc.gov/breastfeeding/data/nis_data/results.html.

[10] Ajzen I. The theory of planned behavior. Organ Behav Hum Decis Process. 1991;50:179–211. 10.1016/0749-5978(91)90020-T.

[11] Montaño DE, Kasprzyk D. Theory of reasoned action, theory of planned behavior, and the integrated behavioral model. In: Glanz K, Rimer BK, Viswanath K, editors. Health Behav Health Educ Theory Res Pract. 4th ed. San Francisco, CA: Jossey-Bass; 2008. pp. 67–96.

[12] Bandura A. Health promotion by social cognitive means. Health Educ Behav. 2004;31:143–64. 10.1177/1090198104263660. 15090118 10.1177/1090198104263660

[13] Perkins HW. The emergence and evolution of the social norms approach to substance abuse prevention. In: Perkins HW, editor. Soc norms Approach Prev Sch Coll Age Subst abuse Handb Educ Couns Clin. Jossey-Bass; 2003. pp. 3–17.

[14] Austen EL, Dignam J, Hauf P. Using breastfeeding images to promote breastfeeding among young adults. Health Psychol Open. 2016;3:2055102916671015. 10.1177/2055102916671015. 35223072 10.1177/2055102916671015 PMC8864535

[15] Duvall S-S. Not simply the breast: media discourses of celebrity, breastfeeding, and normalcy. Fem Media Stud. 2015;15:324–40. 10.1080/14680777.2014.919334.

[16] Foss KA. Perpetuating scientific motherhood: infant feeding discourse in Parents Magazine, 1930–2007. Women Health. 2010;50:297–311. 10.1080/03630242.2010.480905. 20512747 10.1080/03630242.2010.480905

[17] Newell C, Sandoz E, Tyndall I. A pilot study of the impact of brief exposure to images of breastfeeding mothers on attitudes toward mother’s breastfeeding in public. Health Commun. 2022;37:185–90. 10.1080/10410236.2020.1830511. 33019836 10.1080/10410236.2020.1830511

[18] Foss KA, Southwell BG. Infant feeding and the media: the relationship between parents’ magazine content and breastfeeding, 1972–2000. Int Breastfeed J. 2006;1:10. 10.1186/1746-4358-1-10. 16722542 10.1186/1746-4358-1-10 PMC1489921

[19] Foss KA. That’s not a beer bong, it’s a breast pump! Representations of breastfeeding in prime-time fictional television. Health Commun. 2013;28:329–40. 10.1080/10410236.2012.685692. 22746199 10.1080/10410236.2012.685692

[20] Lazalde G, Nakphong M. YouTube as lactation consultant: a content analysis of breastfeeding videos on YouTube. Public Health Rev. 2019;2.

[21] Petit M, Smart DA, Sattler V, Wood NK. Examination of factors that contribute to breastfeeding disparities and inequities for black women in the US. J Nutr Educ Behav. 2021;53:977–86. 10.1016/j.jneb.2021.08.013. 34763821 10.1016/j.jneb.2021.08.013

[22] Schindler-Ruwisch J, Aluc A. The relationship of race and ethnicity to the perception of visual images of breastfeeding mothers. Breastfeed Med. 2022;17:459–65. 10.1089/bfm.2021.0296. 35180357 10.1089/bfm.2021.0296

[23] Adobe. Adobe Fast Facts [Internet]. 2023 [cited 2023 Jun 30]. Available from: https://www.adobe.com/about-adobe/fast-facts.html.

[24] Adobe. Adobe Stock [Internet]. 2022 [cited 2022 Jun 3]. Available from: https://stock.adobe.com/.

[25] Google. Google Sheets: Online Spreadsheet Editor [Internet]. 2022 [cited 2022 Jul 5]. Available from: https://www.google.com/sheets/about/.

[26] Adobe. Edit and Delete Files [Internet]. 2022 [cited 2023 Feb 22]. Available from: https://helpx.adobe.com/content/help/en/stock/contributor/help/editing-and-deleting-files.html.

[27] Adobe. Account and Submission Guidelines [Internet]. 2022 [cited 2023 Feb 22]. Available from: https://helpx.adobe.com/content/help/en/stock/contributor/help/submission-guidelines.html.

[28] Fullilove MT. Comment: abandoning race as a variable in public health research–an idea whose time has come. Am J Public Health. 1998;88:1297–8. 9736864 10.2105/ajph.88.9.1297 PMC1509076

[29] Yudell M, Roberts D, DeSalle R, Tishkoff S. Taking race out of human genetics. Science. 2016;351:564–5. 10.1126/science.aac4951. 26912690 10.1126/science.aac4951

[30] Telles E. The project on ethnicity and race in Latin America. Pigmentocracies: ethnicity, race, and Color in Latin America. University of North Carolina Press; 2014.

[31] Campbell ME, Keith VM, Gonlin V, Carter-Sowell AR. Is a picture worth a thousand words? An experiment comparing observer-based skin tone measures. Race Soc Probl. 2020;12:266–78. 10.1007/s12552-020-09294-0.

[32] IBM Corporation. SPSS. Chicago, IL: IBM Corporation; 2020.

[33] Telles E, Flores RD, Urrea-Giraldo F, Pigmentocracies. Educational inequality, skin color and census ethnoracial identification in eight latin American countries. Res Soc Stratif Mobil. 2015;40:39–58. 10.1016/j.rssm.2015.02.002.

[34] Chapman S, Ryan-Vig S, Cochrane Knowledge Translation, Cochrane UK. Choosing Images for Sharing Evidence: A Guide [Internet]. Cochrane; 2020 Sep. Available from: https://training.cochrane.org/resource/choosing-images-for-sharing-evidence.

[35] Kislinger L, Kotrschal K. Hunters and gatherers of pictures: why photography has become a human universal. Front Psychol. 2021;12. 10.3389/fpsyg.2021.654474. 10.3389/fpsyg.2021.654474 PMC8217823 34168589

[36] Matriano MG, Ivers R, Meedya S. Factors that influence women’s decision on infant feeding: an integrative review. Women Birth. 2022;35:430–9. 10.1016/j.wombi.2021.10.005. 34674954 10.1016/j.wombi.2021.10.005

[37] Frazer RL, Anderson K. Media representations of race, ability, and gender in three outdoor magazines: a content analysis of photographic images. J Outdoor Recreat Educ Leadersh. 2018;10:270–3. 10.18666/JOREL-2018-V10-I3-9051.

[38] Harkness M, Wallace C. Exposing racial bias in midwifery education: a content analysis of images and text in Myles Textbook for midwives. MIDIRS Midwifery Dig. 2022;32:305–10. 10.1101/2021.10.07.21264614.

[39] Rosenstock Gonzalez YR, Williams D, Herbenick D. Skin color and skin tone diversity in human sexuality textbook anatomical diagrams. J Sex Marital Ther. 2022;48:285–94. 10.1080/0092623X.2021.1989533. 34647496 10.1080/0092623X.2021.1989533

[40] Villalobos AVK, Davis C, Turner MM, Long S, Hull S, Lapinski MK. Breastfeeding in context: African American women’s normative referents, salient identities, and perceived social norms. Health Educ Behav. 2021;48:496–506. 10.1177/10901981211014445. 34027709 10.1177/10901981211014445 PMC12750970

[41] Giles F. Images of women breastfeeding in public: solitude and sociality in recent photographic portraiture. Int Breastfeed J. 2018;13:52. 10.1186/s13006-018-0194-5. 30534190 10.1186/s13006-018-0194-5 PMC6282357

[42] Breastfeeding Committee US. Landscape of Breastfeeding Support Image Gallery [Internet]. n.d. [cited 2023 Jun 30]. Available from: https://www.usbreastfeeding.org/photo-project.html.

[43] Killings N. The Melanated Mammary Atlas [Internet]. Lioness Lact. LLC. 2021 [cited 2023 Jun 30]. Available from: https://www.mmatlas.com.

[44] Centers for Disease Control and Prevention. Public Health Image Library (PHIL) [Internet]. n.d. [cited 2023 Jun 30]. Available from: https://phil.cdc.gov/QuickSearch.aspx?key=true.

